# Quality of Life of Older Persons: The Role and Challenges of Social Services Providers

**DOI:** 10.3390/ijerph19148573

**Published:** 2022-07-14

**Authors:** Mihaela Ghenţa, Aniela Matei, Luise Mladen-Macovei, Simona Stănescu

**Affiliations:** 1 National Scientific Research Institute for Labour and Social Protection (INCSMPS), 010643 Bucharest, Romania; aniela.matei@incsmps.ro (A.M.); luisemladen@hotmail.com (L.M.-M.); 2 Research Institute for Quality of Life (ICCV), 050711 Bucharest, Romania; simona_vonica@yahoo.com

**Keywords:** social services, older persons, quality of life, quality of care

## Abstract

Considering the growing number of older persons, ensuring the quality of life of them, as well as the social services designed for this population category, has become more and more important. Especially in the case of dependent older persons, social services are essential components, as they contribute to a better quality of life. The aim of this paper was to examine the perspectives of social services providers for older persons with respect to their role and the challenges encountered in ensuring the quality of life of older beneficiaries. In order to answer our objectives, we employed a qualitative methodology, using the focus group method to collect information from social services providers (both residential and home care). Multiple factors are related to a good quality of life in old age: some are related to individual characteristics, while others are related to the provision of services. The provision of quality social services that adequately respond to the needs of beneficiaries contributes to increasing the degree of independence and maintaining the physical and mental health of dependent older persons.

## 1. Introduction

The share of the population aged 65 and over has continuously increased in recent years in Romania, from 17% in 2015 to 19.3% in 2021, and that of people aged 80 and over has increased in the same period from 4.1% to 4.8% [1]. Romania is expected to have 4.1 million people 65 years or older by 2035 and 4.5 million by 2050, accounting for 23.3% and 27.7% of the total population, respectively [2].

In 2019, life expectancy in Romania at age 65 for men was 14.9 years (compared to 18.3 years in the European Union) and 18.6 years for women (compared to 21.8 years in the European Union). However, in 2019, healthy life expectancy at age 65 was lower for both sexes: 6.7 years for men (10.2 years in the European Union) and 6.5 years for women (10.4 years in the European Union), resulting in a greater need for adequate social services to meet higher care needs [1]. At the European level, almost 50% of people 65 years or older reported long-term restrictions in daily activities, whereas more than two-thirds reported physical or sensory functional limitations [3]. Nevertheless, Romania allocates only 0.31% of its GDP to long-term care, compared to the 1.6% EU average [1]. Providing long-term, good quality care services that are integrated, people-centered, and properly managed is the right step for ensuring healthy lives and well-being in old age [4]. The difficulties older persons experience have been exacerbated due to the COVID-19 pandemic. Older persons have been recognized as the most vulnerable category of the population, and the need for policy measures directly targeted to them has thus became more necessary in this period [5]. 

Considering the current situation, the aim of this article is to examine the perspective of social services providers with regard to their role and the challenges encountered in ensuring the quality of life of older beneficiaries. The objectives of the research were: (1) to explore the opinions of social services providers with regard to their contribution to the quality of life of beneficiaries; (2) to highlight the views of social services providers in terms of their contribution to quality of care (autonomy and independence of older persons, freedom of decision, control, sense of security and respect for privacy, communication, and social interactions); and (3) to identify the difficulties experienced in the services delivery process.

### 1.1. Social Services for Older People Living in Romania

In Romania, social services addressed to older people are provided by both public and private social services providers (associations, foundations, cults recognized by law), and are offered on a residential or non-residential basis. The first category of services includes care and assistance centers for older people, such as nursing homes, respite/crisis centers, and sheltered housing, as well as residential care and medical-social assistance centers. The second category of services includes home care units and day centers for older persons, such as day and recovery centers and day centers for socializing and leisure. 

Within the current social protection system, older people have the right to social assistance in relation to their social and medical status and the economic resources (income, assets, etc.) they possess. The legal framework stipulates that decisions regarding admitting an older person to a residential center are made considering the following priority criteria: if the person requires special permanent medical care, which cannot be provided at home; if the person cannot manage on his/her own, is without legal supporters, or is unable to fulfill their obligations due to their health or economic situation and family responsibilities; if the person has no home and no income of his/her own. However, home care and keeping older people in their living environments are much more suitable for those in difficulty, with these measures having a multitude of benefits [6]. 

The field of social services is still poorly developed at the national level, with the number of accredited providers and licensed services being quite small compared to existing needs. The geographical coverage of these services is not balanced, with a higher concentration of providers in urban areas and in more developed counties [5,7]. The main source of funding for social services is the local budget, so the number and quality of social services for the elderly are highly dependent on the financial capacity of the administrative-territorial units [8]. The financing of social services is also derived from the contribution of the beneficiary and/or their family, from the state budget, as well as from other sources. The local public administration authorities finance the public social services under their authority. The rest of the social services are financed by contracts awarded through public procurement or by grants or subsidies. The standard cost of personal home care services varied in 2020 between 3225 EUR (15,600 RON) and 6450 EUR (31,200 RON) per year, and of residential social services for the elderly between 5321 EUR (25,738 RON) and 10,451 EUR (50,554 RON) per year, according to the degree of dependence in which the person is included [9]. In the same year (2020), the annual average pension for old age was 4061 EUR (19,644 RON) [10], and 24.5% of older persons aged 65 and over—22.3% of pensioners—were at risk of poverty [1].

Access to social services for older people is essential due to their specific needs, and offers them a dignified life. The quality of social services provided to these people is reflected in their quality of life. It can be stated that the quality of life of older people is closely linked to the social protection system and the quality of social services they receive [11].

### 1.2. Quality of Life of Older Persons in Home and Residential Services

Quality of life is a complex, multifaceted concept, having several components that interact simultaneously: objective, subjective, macro-societal, micro-individual, positive and negative components [12,13,14]. The macro- (societal, objective) component refers to the roles of income, employment, housing, education, and other living and environmental circumstances, while the micro- (individual, subjective) component considers perceptions of overall quality of life, individuals’ experiences and values, and related proxy indicators such as well-being, happiness, and life satisfaction [15].

Despite the growth in scientific inquiry regarding quality of life, there is no generally accepted definition of the concept or a way to measure it [12]. Quality of life in old age and well-being are more often associated with good health and functional ability, a sense of personal adequacy or usefulness, social participation, intergenerational family relationships, the availability of friends and social support, and socioeconomic status [16,17,18]. However, quality of life in old age often differs between various groups of older people [19,20]. For example, there are differences in perceptions between older people living in the community and older people in institutional care. The former values more social integration and the latter, the quality of the environment. Additionally, for older people in institutional environments, their significant priorities are in control over their lives, the structure of the day, a sense of self, activities, and relationships with staff and other residents [21].

A number of studies have focused on diverse quality of life issues of older people in residential care homes. The body of literature reveals that quality of life of these persons is greatly determined by their independence, individuality, and autonomy. It is also influenced by the quality of residential care and facilities [22]. Duncan-Myers and Huebner [23] identified a strong positive correlation between improved quality of life of older people and increased frequency of choices available to them in self-care and leisure activities. An important aim is to help older people in residential care to maintain self-control of their lives and care. People who have more independence in performing activities of daily living (ADL) and instrumental activities of daily living (IADL) have reported better quality of life [24]. Studies have also found a significant impact of social health on the quality of life of older people in residential care homes. Social factors, including socio-economic status, perceived social support from caregivers, and frequency of interactions with family have been reported as predictors of quality of life of older people in residential care facilities [25]. Institutionalization is a stressful experience for older people, having a detrimental impact on their physical and psychosocial well-being. Environmental modification in the sense of promoting a home-like physical and social atmosphere, autonomy, and individuality is seen to be beneficial for older people in residential care homes [22]. 

Research has also focused on the analysis of the quality of life of older people receiving community-based care. Large-scale studies indicate that the level, maintenance, and development of high-quality social support networks contribute to improved quality of life [26,27]. Older adults’ social networks could provide access to support and assistance from family members, neighbors, friends, and service providers. This type of support is essential and is related to older adults’ perception of control and improved well-being [27]. Studies detail that social participation opportunities and provision of care services that meet older adults’ needs are associated with higher quality of life [28].

The growth in the older population has contributed to an increased demand for social services that are cost neutral and that maximize the quality of life of beneficiaries [29,30,31]. Quality of life has become a standard measure of long-term care services outcomes [29,30,31,32,33]. Care homes could be an important part of caring for older dependent people. Despite this, across all cultures, up to 90% of older adults prefer to remain in their own homes for as long as they can [34]. The trend at the European level is to move from an institutional care system to a system that provides care for older people in a more familiar environment, i.e., at home. Existing studies have found that older people enjoy a higher quality of life if they remain in their home receiving the care they need than if they live in a residential care institution [22,35,36,37,38]. 

Other studies have not found differences in the quality of life between institutionalized older people and non-institutionalized ones [39]. At the same time, some studies have shown positive aspects of institutionalization, such as adherence to pharmacotherapy, improvement in social life, easing of depressive symptoms, and participation in leisure activities that benefit locomotion [40]. 

Irrespective of the type of social services, the quality of life of dependent older people is a difficult issue to manage, especially where there is no family support in the physical proximity of the older person, as in the case of transnational families [41], which changes the paradigm of understanding the family based on the idea of co-residence and physical unity [42]. Migration causes the spatial separation of families, with the care of dependent elderly people left behind in their home countries being a real challenge for members of transnational families, as shown by the studies conducted on this topic [43,44,45,46,47]. Although migration does not make mutual care obligations and expectations disappear, members of transnational families usually involved in different types of transnational care of relatives who remain in their countries of origin encounter difficulties in fulfilling their roles in the absence of a well-developed long-term care system in the home country. The forms of care provided by members of transnational families for dependent relatives left behind also depend on cultural norms that define the duties of the younger generation towards senior family members.

## 2. Materials and Methods

### 2.1. Conceptual Framework

Studies in the field have identified, as the main determinants of quality of life in old age, psychological/emotional aspects (life expectations, acceptance of physical dependence), health (mobility, functional autonomy), social participation and social support (involvement in social, cultural, religious activities), social capital (relationships with family, friends, other residents), and the feeling of security [18,48,49,50,51,52,53]. A qualitative methodology was developed starting from this conceptual scheme that refers to general determinants of quality of life in order to generate information on the specific determinants of the quality of life of dependent elderly people in Romania. The qualitative methodology offers a research tool that enables the understanding of the complexity of the phenomenon of quality of life of dependent elderly people in Romania, measured from the perspective of social services providers involved in managing the phenomenon [54,55]. 

### 2.2. Study Area

The qualitative methodology developed by the research team was applied in eight focus groups organized in each of the eight development regions of Romania, participants in these focus groups being public and private providers of social services with and without accommodation for dependent older persons. According to the nomenclature of social services in Romania, accommodation services include social services provided in homes for the elderly—8730 CR-VI; respite centers—8730 CR-V-II; sheltered housing—8730 CR-V-III; services without accommodation, which include social services provided in day care and recovery centers—8810 CZ-VI; and home care units—8810 ID-I. The situation of participation in regional focus groups is presented in Appendix A. 

### 2.3. Method, Recruitment, Data Collection, and Analysis

The authors developed a focus group guide within this qualitative methodology, with the conceptual dimensions of the research tool relying on scientific literature in the field. The focus group technique enables, through interactions with respondents, the development of a discussion about a topic [48,52,53]. Analysis of the information gathered enables the identification and study of the social meaning of the studied topic [7,56].

The study was carried out between May and June 2019. As the data collection occurred before 2020, the analysis provides a picture of the determinants of the quality of life of older persons in social services just before the COVID-19 pandemic. The sample size took into account the aim of the research, the sample specificity, and analysis strategy [57] (p. 402). Theoretical sampling was used in the selection of participants in the focus group, the main selection criterion being the relevance of the investigation unit for the research objectives. In the case of our research, the relevance of the investigation units was given by the information they have about the quality of life of the elderly [58]. The public and private providers that participated in the focus groups were selected from those included in the Single Electronic Register of Social Services provided by the Romanian Ministry of Labor and Social Justice (MMJS) in January 2019.

The tool used (focus group guide) addressed the following topics: (1) quality of life of dependent older people in Romania; (2) autonomy and independence, quality assurance of care; (3) freedom of decision, control, sense of security, and respect for privacy; (4) communication and social interactions, social participation/activities carried out (Table 1). 

Each focus group session lasted 60–90 min, was conducted by a moderator and an observer, and was digitally recorded with the participant’s oral consent [53,55]. All the social services providers for the elderly received an e-mail with an invitation to participate in the research. Social services providers willing to participate in the research appointed a person to participate in a focus group. Following this, informed consent was obtained from all participants of each focus group, and this consent was audio recorded.

The research team used the qualitative research software NVivo12Pro to record and manage the data (Table 2). After all the focus groups’ data were transcribed, the transcripts were analyzed following the principles of thematic analysis, which reveals the content of and meanings behind patterns (themes) present across entire data sets [59]. The coding was conducted in two phases. In the first phase, two researchers assigned independently all the responses to each theme of the focus group guide. Inconsistencies in text coding between the two researchers were reviewed and refined after another revision of the transcripts and audio recordings. In the second phase, analyses of results were jointly conducted by all authors. As the participants of the focus group discussions were professionals nominated by each nationally accredited social services provider for the elderly that agreed to participate in the research; therefore, a good degree of credibility of the results can be assumed. Text passages are presented in the Results section by type of service and development region (Bucharest Ilfov, BI; Centre, C; East, E; Nord West, NW; South Muntenia, SM; South East, SE; South-West, SW; West, W).

## 3. Results

### 3.1. Quality of Life of Dependent Older People in Romania

#### 3.1.1. Factors (Positive/Negative) That Influence the Quality of Life of Dependent Older People

The quality of life of dependent older people was defined by social services providers as meeting the daily needs of dignified care and proper access to various forms of support or to social services. Quality of life implies the ability of each person to satisfy his/her needs and to have access to medical services and social activities adequate to their health status.

*“In general, the quality of life requires at least a satisfactory degree of fulfillment of a person’s specific needs, in our case semi-dependent or dependent older people.”*

(Residential care provider, BI.)

*“First of all, the older person must have a permanent connection with the family doctor, the caregiver must take care of the older person’s scheduling and medical assistance. At home, personal hygiene is needed as much as possible, the older person must have a social life, if possible.”*

(Home care provider, SE.)

Social services providers considered that the main positive factors with an influence on the quality of life of older dependent persons that are receiving home or residential care were related mainly to income level, health status, and access to healthcare services, while the negative factors were related to health status, the ability to move independently, and relationships with family members (Table 3). 

In the case of home care services, the social services providers mentioned the risk of inadequate care despite the good intentions of the caregiver. Residential care was considered to be able to contribute to a better quality of life for dependent elderly people, as it facilitates access to adequate medication, staff, and medical care despite the social stigma associated with the institutionalization of older family members. The main disadvantage mentioned for home care services was the inability to respond positively to all the needs that an elderly person in home care has.

*“* *I think that the whole of society needs an education in this regard, which is why those who are at home suffer. Because of some mentality, a poor education, to do things in such a way as to reconcile society more than the older person in need. They come and say: I can’t take my mother or my father to a nursing home! What will my neighbors say?”*

(Residential care provider, C.)

*“* *They should have access to quality medical services, including an ambulance that fails to arrive.”*

(Home care provider, BI.)

In the case of residential social services, occupational therapy can positively contribute to a better quality of life for the elderly. It involves the provider access to different types of activities that give the beneficiary a feeling of freedom and sense of utility, and that fosters connections with the previous life and occupations of the beneficiary.

*“* *Occupational therapy helps a lot. The vast majority of the elderly who, at some point, end up accessing residential social services, want to continue the activities that they were taught and used to do.”*

(Residential care provider, SM.)

Communication and the involvement in different social activities improve the quality of life of older dependent persons and reduce social isolation in old age, but the scarcity of occupational therapists, physiotherapists, and psychologists is common with both residential and home care providers, and it is felt especially in small towns and rural areas. 

For beneficiaries that have good relationships with family members, the effects are positive in regard to their emotional status and sense of belonging.

*“* *There were cases where grandchildren, uncles, and aunts came. The fact that they came is good. Older persons may have dementia, but you can talk to most of them and they know they have a family. It’s something that improves their daily mood.”*

(Residential care provider, W.)

Intergenerational activities provide the opportunity for older dependent persons to feel part of the community again, to exchange experiences and knowledge and to feel valued.

*“Collaboration between generations is very important. Older people enjoy exchanging information with younger people, children, and teenagers.”*

(Home care provider, C.)

In terms of negative factors, the quality of care is limited by the available resources of providers and the time that human resources have at their disposal.

*“And the services are not easy to provide, the nursing job is hard, difficult … it requires a lot of physical effort and mental consumption … and you end up being over-saturated in all respects … burnout.”*

(Residential care provider, NW.)

Last but not least, bureaucratic requirements directly diminish the ability of providers (irrespective to where the services are provided) to respond to the needs of beneficiaries.

*“* *I have to be available if the institution hires me and has 300 beneficiaries. I, a social worker, have to be available to prepare the files and you don’t want to know what a medical-social file means to be prepared, right? Or, when the ministry comes for an inspection and controls the paperwork, it’s extremely messy. A bunch of documents that from my point of view, forgive me … are made in vain. I don’t see the point … except that we cut down trees and throw them away, and the beneficiary receives what? My frustrations, his unresolved frustrations … […] The time allotted to the beneficiary is then shortened.”*

(Home care provider, BI.)

Other negative factors pointed out by the social services providers were related to the difficulty of meeting the quality requirements set in the national standards of quality for social services, the lack of adequate financial resources, the lack of specialized staff, and the changes to the legal framework in the field of social assistance. 

#### 3.1.2. The Impact of Social Services on Quality of Life 

The provision of appropriate social services that adequately respond to the needs of beneficiaries, directly contribute to an increased degree of independence and maintenance of their physical and mental health was reported as being beneficial.

*“The existence of the social service itself is beneficial for dependent and semi-dependent older persons.”*

(Home care provider, BI.)

Cases of significant improvement in health following residential care were reported. Meanwhile, for home care provided by untrained informal carers or family members, cases when the beneficiary’s level of dependence was accentuated despite good intentions were noted.

*“We had positive results when they came on a cart or in bed and then they managed to walk without a cane.”*

(Residential care provider, SM.)

In the case of home care, the lack of human resources and services (e.g., personal hygiene), difficulties in complying with medication on time by the beneficiary, and the positive impact of social visits to the dependent elderly person were also mentioned. 

Social services providers emphasized the importance of assessing the quality of social services provided in order to improve, diversify, and adapt them as best as possible to meet the needs of beneficiaries. Consultation with both beneficiaries and their relatives were taken into account. However, the bureaucracy related to admission into social services was cited as a barrier. Another problem faced by residential centers is the difficulty of managing the presence in the same home of dependent but mentally healthy elderly people and those with dementia (including persons over 20 years old diagnosed with autism). 

Social services providers appreciated that a first direction is the continuous improvement of the quality of social services offered through individualization instead of diversification. Financial incentives for existing staff and the identification of ways to attract a young and skilled labor force could cover the necessary human resources.

Other areas mentioned were collaboration with medical staff, the local community, and volunteers (including older persons) and the development of national public information and awareness campaigns about the social services available for dependent older people. The possibility to visit residential centers and access direct information could allow for the overcoming of stereotypes related to the abandonment of older family members in residential settings. The development of such national education programs would allow for the provision of social services in residential centers or at home in time, preventively not at the moment when a person’s health status is far too deteriorated to be remedied. 

The impact of social services on the quality of life of dependent older people is constantly measured through questionnaires, observations, activities carried out, and discussions whenever necessary. Measuring the influence of the quality of care on the quality of life of beneficiaries is requested within the national quality standards. In the present study, social services providers were concerned with overcoming the formal level of completing the questionnaire and identifying those issues that may contribute to a better quality of life of beneficiaries. In this respect, the information obtained from the beneficiaries was correlated with that obtained from the assigned staff and subjected to discussions in the team meetings. 

### 3.2. Autonomy and Independence; Quality Assurance of Care

Within this dimension, the beneficiary’s ability to carry out basic activities of daily living, dependency, and mobility and their level of vitality were taken into account.

#### 3.2.1. The Role of Social Services in Maintaining the Independence of Older People

Social services providers reported that concerns for the preservation of the functional autonomy of beneficiaries was the main aim of their activity. A first obstacle in this regard is the number of and the training of the human resources involved. 

Social services contribute to the improvement of the quality of life of older person, as they cover activities of daily living that can no longer be carried out by the person. Moreover, in the case of residential centers, an older person has access to adequate health care and specialized staff trained to support the older person in maintaining a level of autonomy and independence.

*“* *I have found that at home, even if people are hired to take care of them, they are untrained. We have seen cases in which they have turned a semi-dependent person into a completely dependent person. Why? Because it’s very convenient to change a diaper. That person should stay in bed, calm, quiet and why waste time with mobilization?! And so it turns a semi-dependent people into a totally dependent people, through the care they provide. Their degree of independence has increased because we do physical therapy, we do treatment, we monitor, plus they socialize very well and there are also rules that they would not follow at home. At home they are very comfortable, at home they are used to taboos. Here, they are willing to accept rules that we are aware of and convinced that are to their advantage.”*

(Residential care provider, SW.)

In the case of residential services, due to the advanced state of dependence at admission, in many cases, the provider does not necessarily aim to improve the autonomy of the person through the services they offer, but rather to lower the process of physical or mental degradation, while taking into account the pathology of each person.

*“When they get to a residential center, they find themselves cared for, nothing bad can happen to them. After a certain period of time of accommodation, they enter into a certain routine and everything is okay for them, no matter how sick they may be, especially in cases of dementia. They feel protected that nothing can happen to them, there is immediately someone next to them who helps them when needed, they enter a certain stage, the evolution is very slow, with the exception of the compensations that appear later due to other reasons.”*

(Residential care provider, SW.)

The involvement of beneficiaries in maintaining a degree of functional autonomy depends on their mental state, their degree of awareness with regard to their health status, the accessibility of the living environment, their feelings of trust in their care staff and their existence, as well as the involvement of family members.

Social services providers reported that the majority of beneficiaries were concerned with maintaining functional autonomy, especially in the case of home care beneficiaries where there are not always people available to help. These beneficiaries were characterized as healthier, more open to interaction, balanced, smiling, well-disposed, and able to decide on their own whether to move to a residential center.

Residential social services providers mentioned a number of autonomous activities that the elderly carry out: personal hygiene, serving meals, caring for plants, participating in various activities within the residential center (library, meetings) and community (church). In the case of those cared for at home, the following were mentioned: small walks in the park, shopping, and going to the market. 

In addition to the social services provided, participants mentioned other types of services, which the beneficiaries request in order to maintain functional autonomy: prostheses, orthoses, hearing aids, and dental services and implants. 

#### 3.2.2. The Role of Dependent Older People/Legal/Conventional Representatives in Improving the Quality of Care

Beneficiaries, their families, or other legal representatives may influence the quality of care. In some cases, family involvement has positive effects on the well-being of the dependent older person, and these benefits were pointed out by both home care and residential services providers.

*“The efforts of the team no longer lead to the same result, to the same quality of service, to the same degree of satisfaction for the beneficiary and the same quality no longer reaches the beneficiary.”*

(Residential care provider, SM.)

Participants in focus group discussions reported that semi-dependent older people are more interested in maintaining their level of autonomy. In the case of people with various cognitive pathologies, their interest in maintaining good physical health was reported as being rather low.

*“It depends on the disease and how aware they are of this. Often there are certain relatives who make the decision on their behalf, especially in cases of dementia…. Many do not have the necessary will. It matters a lot! If they don’t get involved, you have no results. You need to find the ways to show them that what you are doing is for their own benefit! It takes a lot of patience and involvement!”*

(Residential care provider, SW.)

Maintaining the autonomy and independence of beneficiaries is a major concern among older people who are in a better emotional state, whether they receive home care or residential care. The participants mentioned that those persons who have a daily life schedule, with regular activities, are more interested in maintaining their autonomy and independence. The involvement of family members in providing services helps to improve the older person’s emotional state and is often essential.

*“I say that it is very important to involve the family in everything you do. I had people who were in bed and benefited from this collaboration between the family and the organization.”*

(Residential care provider, C.)

### 3.3. Freedom of Decision, Control, Sense of Security, and Respect for Privacy

#### 3.3.1. Freedom of Decision Regarding the Type of Care 

Freedom of decision is limited by the degree of dependence (physical and mental) of each person, the social services available, the area of residence (rural or urban), and last but not least, the available financial resources.

*“And mental health, depending on each individual, the social situation in which they are… And secondly, the range of social services within the region or within the community. In rural areas they are non-existent.”*

(Residential care provider, SM.)

Freedom of decision is also limited by the lack of information about available services that an older person can access.

*“Unfortunately, this freedom of decision does not exist as it should, because even if they want a specific service, they want a specific care … they can’t find it or don’t know where to look for it.”*

(Home care provider, BI.)

Family members also perform an important role in choosing the social services for dependent older people, as the costs associated with such services are often supported by them.

*“There are extraordinary differences and the family says: up to here, up to the money.”*

(Residential care provider, SM.)

According to social services providers, the decisions of beneficiaries are respected, and they take into account the characteristics of the social services provider. However, the spectrum of services from which dependent older people can decide is limited. 

According to the social services providers, collaboration with beneficiaries and family members is vital in ensuring the quality of care, irrespective of where the social services are provided (at home or in a residential setting). Reduced understanding of the limits of social services provision both by beneficiaries and relatives leads to unrealistic expectations and even tensions. 

The quality of life of beneficiaries is also influenced by the way in which connections with the human resources involved in care are built. The skills reported as necessary to perform the tasks of care were professional skills and the ability to manage difficult situations. Due to the specific nature of caring for dependent elderly people, some employees decide not to stay (even after care experiences abroad) and some volunteers do not continue their activity. Another problem is the ageing of the care staff, which reduces their physical capacity to manage certain situations that appear during the care process. 

#### 3.3.2. Safety of Social Services Provided

According to social services providers, when beneficiaries consider that their personal safety is endangered, written complaints are addressed to the authorities in charge. Social services providers also emphasized their compliance with standard protocols and procedures (strict guidelines for medication and care, evaluation, and monitoring visits). A positive image of care staff in the eyes of beneficiaries is important in a good care process.

*“Procedures should be followed ad literam. If you have a procedure, you follow it. […] Where there is a lack of procedure, there is chaos.”*

(Home care provider, BI.)

With regard to the security felt by beneficiaries during the provision of services, the order and the daily routine of care can provide them with a feeling of security. Safety in the provision of social services requires the existence of qualified staff, continuous collaboration between the members of the provider’s team, and control and supervision over the way in which the care is provided by the staff.

*“First of all, continuous surveillance. They keep going to the sick persons and observe them. I don’t know how home caregivers cope because the condition of an elderly person can be so misleading. They might be fine now and then fall on their feet in five minutes.”*

(Residential care provider, C.)

#### 3.3.3. Respect for Privacy 

Respect for the privacy of a dependent elderly person is important regardless of where the services are provided, the provider having the responsibility to ensure the training of staff in this regard. A first step in this delicate process is to understand the meaning of privacy for a dependent person. The patience and training of the care staff influence the way in which beneficiaries perceive that their privacy is respected.

*“You shouldn’t ask him more than he wants and can give you as information. You have to have some limits. If he doesn’t want to tell you more, you don’t insist upon asking annoying questions.”*

(Home care provider, BI.)

Representatives of residential social services and home care providers reported that care for the safety of a beneficiary is ensured by following the standard procedures of caregivers. Respecting the privacy of an elderly person implies respect for his/her religious beliefs, and in the case of home care services, it is important to respect the wishes of and restrictions imposed by the person during the provision of services.

*“The person’s privacy package, the bedside table where the person has their personal belongings, in the immediate vicinity of the bed there are icons or photos from their youth, indicating their desire to arrange their own corner.”*

(Residential care provider, SM.)

### 3.4. Communication and Social Interactions, Social Participation/Activities Carried Out

#### 3.4.1. The Importance of Social interaction Activities 

Providers of social care services for dependent elderly people reported that they appreciate that communication and involvement in social activities are important. In the case of residential social services, the frequency of social activities depends on a number of factors, such as the availability of human resources, the occasion for which they are organized, available income, the health state of beneficiaries, and their willingness to engage. In the case of home care services, involvement in social activities depends on the level of autonomy of each person. Carrying out such activities changes the mood of beneficiaries and maintains good emotional well-being.

*“They want to have someone to talk to, to have someone to socialize with. Not to feel alone, not to fall into a depression, not to feel insignificant in this world.”*

(Residential care provider, NW.)

Social services providers noted that care staff are ready to support elderly beneficiaries’ involvement in social activities. The involvement of human resources depends on the level of professional training and of personal skills. Compliance with internal regulations supports the development of care. From this perspective, the high load of care staff and the involvement of volunteers were mentioned.

Residential social services providers mentioned a wide range of activities: art courses (painting on canvas and glass), sports activities (dancing, table tennis, cycling in the yard), activities organized within the center (folk performances, marching bands, songs and poetry, counseling on various topics, meeting with the priest, visits to the chapel, and involvement in culinary activities), other leisure activities (choir, karaoke, rummy, chess, backgammon, watching movies, prayer in the chapel of the residential center), and trips outside the home (walks, pilgrimages, going to church, watching shows or movies, short visits to home or relatives). Participation in mountain or sea trips depends both on a beneficiary’s ability to travel independently and logistical aspects specific to transport (e.g., covering the costs involved). In the case of beneficiaries cared for at home, they may be accompanied to carry out various activities. Within the focus group discussions, there were representatives of social services providers who stated that they managed to collaborate with different private organizations in organizing trips for dependent older people.

Regarding the interaction of dependent elderly people with other categories of people outside the organization, social services providers mentioned: relatives, friends, community members, and neighbors (in the case of rural areas). 

#### 3.4.2. Factors Limiting the Social Participation of the Elderly

In the opinion of social services providers, the factors that limit the participation of dependent elderly people in social activities are: the existence of different health problems (including depression), their limited physical capacity, their previous social status, their previous lifestyle, their interest in involvement in social activities, the characteristics of those with whom they would interact, the attitude of the community towards them, their income level and, last but not least, solving the various logistical aspects related to the organization of such activities (ensuring private transportation means, difficulties in accessing accessible public transport means, availability of staff, etc.). Representatives of social services providers emphasized the importance of permanent supervision when traveling outside the center in order to avoid walking difficulties and falls. Often, the authorized attendant is a family member or a trusted person who can ensure the physical safety of the elderly person while traveling.

#### 3.4.3. The Attitude of the Elderly towards IT Technology

Regarding the attitude of dependent elderly people towards modern means of communication, social services providers reported that beneficiaries are interested in using the Internet and electronic devices related to the field of information technology. Communication and social applications (e.g., Skype, Facebook, email, WhatsApp) are used on various devices (e.g., smartphones, tablets, laptops, and computers). Beneficiaries’ families perform a major procurement and learning role in older people’s accessing and using modern means of communication. Older persons communicate with family members (especially when their children are abroad), receive photos, read the press, search for various information on the Internet, and shop online. Computer rooms are available in some centers. However, not all elderly people are technologically connected in this way. Those who have various mental illnesses are neither interested in nor able to access IT technology. One of the beneficiaries of social services at home interviewed had only a landline phone. Difficulties were also mentioned regarding the use of mobile phones (e.g., abandonment or blocking of telephones, loss of chargers, etc.), cases in which relatives call the care staff for help. 

## 4. Discussion

### 4.1. Limitations of the Research

Some limitations of the present study can be highlighted. The main limitation was that it explored the meaning and the determinants of the quality of life of older persons from the perspective of providers, reflecting only one facet of the issue. Another limitation of the study was that providers of residential services were greater in number compared to home care providers, and thus a less comprehensive picture for understanding the influences of social services on the quality of life of beneficiaries resulted. However, this study contributes to a better understanding of the role of social services providers in ensuring the quality of life of dependent older beneficiaries of social services, and these results could also serve as an evidence base to improve policies regarding older persons. 

### 4.2. Suggestions for Services and Policy 

This study presents the roles involved and challenges encountered in ensuring the quality of life of older beneficiaries. Our findings revealed aspects related to the objective quality of life of older dependent persons, quality of care, freedom of decision, control, sense of security, respect for privacy, the role of communication and social interactions, and social participation/activities.

With respect to the *quality of life*, previous studies [60] outlined objective measures of quality of life that support social policies and programs (and thus a better adequacy of the social services to meet the needs of older beneficiaries), as they capture the effects of such interventions. Other studies [61] argued that objective measures do not reveal the individual perspective. However, the assessment of quality of life cannot remain a purely subjective matter, especially when it is used in a particular social policy context [12,56,62], being one of the most important objectives in caring for older persons [63]. Quality of life of dependent older persons is related to the ability of each person to be able to satisfy their needs and have access to social and health services; it also includes attention to their emotional state. 

Multiple *positive factors* were pointed by the participants as being related to a good quality of life in old age: an adequate level of income, a healthy lifestyle, access to necessary and affordable healthcare services, good relationships with family members, and involvement in social activities. Participants highlighted certain advantages of residential care compared to home care, for instance, despite their good intentions, family caregivers do not possess the knowledge to provide adequate care. These findings suggest the need for the training and education of caregivers and family members using various methods (video, the Internet, etc.) to inform and train them on how to perform caring tasks, as other studies related to older persons have concluded [64]. Existing studies have highlighted that the COVID-19 pandemic increased the care burden of older people’s family members [65,66]. In our study, residential care was found to contribute to a better quality of life for dependent elderly people, as it facilitates access to adequate medication, staff, and medical care. In other studies, the results suggested that elderly residents are more likely to experience a deterioration in quality of life due to changes in their living conditions, impaired health, reduced functional autonomy, and decreased social interactions [22,67]. 

*Factors with a negative impact* on the quality of life relate to the physical and mental health of older persons, the characteristics of their environment, and the limited availability of social services, especially in small towns and rural areas. Adequate and trained social and healthcare professionals are essential to ensure the quality of care and to prevent the risk of burnout or abusive care practices in caring for the elderly. Our findings are consistent with studies that suggest the negative impact of burnout on the level of stress of staff [68], as well as the quality of care [8,69]. 

The providers’ preservation of the functional autonomy of beneficiaries was reported as the main aim of their occupation, and this implies both the effort of professionals, beneficiaries, and family members. The influence of the quality of care on the quality of life of beneficiaries is under constant monitoring, and the involvement of older persons and families in the care process is a constant measure for the quality of services delivered (at home or in a setting), as well as for the quality of life of beneficiaries. The process of service delivery becomes an important indicator for outcomes in terms of satisfaction with quality of life of beneficiaries [11,70].

*Communication and social participation* improve the quality of life of older dependent persons and reduce social isolation in old age, but the scarcity of occupational therapists, physiotherapists, and psychologists is common to both residential and home care providers. The results in this study are consistent with previous national and international studies [11,63], according to which social participation has proven to be an important explanatory factor for the quality of life of the elderly. Providers argued that social interactions with family and the community maintain and improve the emotional well-being of older persons, as other studies proved [71,72].

## 5. Conclusions

This study shows that quality of life of older beneficiaries of social services is an important aspect that gives sense and meaning to social services provision for older persons. Our results point to the convergent opinions of providers with respect to the understanding of the quality of life of older dependent persons. Multiple positive factors were pointed out by the providers as being related to a good quality of life in old age: some of the factors are related to individual characteristics, while some of them are related to services provision. Access to necessary and affordable healthcare services is a factor that positively impacts the quality of life. The provision of quality social services that adequately respond to the needs of beneficiaries, increase their degree of independence, and maintain their physical and mental health is also vital. Quality assurance is an important aspect that providers take into account in the provision of social services, and the participants in the qualitative research pointed to some factors that negatively affect this provision (lack of human resources, bureaucracy, etc.). Social services play an important role in maintaining the autonomy of older beneficiaries, and the participating providers stressed that the involvement of beneficiaries and families is very important in this regard. Different opinions in regard to the impact of social services on quality of life of older persons were revealed by home care and residential providers. 

The results of our research conducted among social services providers highlight the need for a fundamental change in the construction and governance of the national system of social services for older persons. This change must take into account the existence of multidisciplinary teams, continued investment in the workforce, better public allocation of resources for social services, finding innovative ways to attract and to maintain the young and specialized workforce (beyond financial motivation, and especially by providing concrete prospects for professional development and increasing professional prestige of the social work profession), and development and diversification of the available social services, especially in rural areas and in communities with high migration rates. All these factors could improve the overall functioning of the social assistance system for older persons and, most importantly, the quality of life of beneficiaries. However, further research should evaluate, on a periodic basis, the quality of life of older persons receiving social services, as well as the effects of high migration flows of labor forces on the older population left behind, and the results should substantiate subsequent improvements of the social policy for this age group.

## Figures and Tables

**Table 1:** Dimensions and indicators operationalized in the focus group guide.

Dimensions of Focus Group	Indicators
Quality of life of dependent older people in Romania	1. Factors (positive/negative) that influence the quality of life of dependent older people 2. The impact of social services on quality of life
Autonomy and independence, quality assurance of care	1. The role of social services in maintaining the independence of older people 2. The role of dependent older people/legal/conventional representatives in improving the quality of care
Freedom of decision, control, sense of security, and respect for privacy	1. Freedom of decision regarding the type of care 2. Safety of social services provided 3. Respect for privacy
Communication and social interactions, social participation/activities carried out	1.The importance of social interaction activities 2. Factors limiting the social participation of the elderly 3. The attitude of the elderly towards IT technology

Source: developed by authors.

**Table 2:** Phases of data analysis.

Phase	Analysis	Means
A	Data coding of transcripts—focus group discussion data (*n* = 8)	NVivo 12 Pro
B	Thematic analysis of transcripts—focus group discussion data (*n* = 8)	NVivo 12 Pro

Source: developed by authors.

**Table 3:** Factors influencing the quality of life of older dependent persons.

Positive Factors	Negative Factors
A decent level of income	Health status deterioration (especially mental)
Maintaining as good as possible health status (physical, cognitive, and emotional)	The loss of the ability to move independently
Access to necessary and affordable healthcare services (including ambulance service, visits from the family doctor or several visits per month to the family doctor).	The lack of age-friendly houses and buildings (e.g., residential buildings without elevators)
Care for nutrition adequate to health status	The lack of relatives or the distance to family members
A good relationship with family members; involvement in social activities (including intergenerational activities)	The weak involvement of the family (especially from the emotional point of view) in the care process

Source: developed by authors.

## Data Availability

Not applicable.

## Appendix A

**Table A1:** Situation of social services providers who participated in the regional focus groups.

Development Regions in Romania	Representatives of Social Services Providers	
	Social services with accommodation	Social services without accommodation
Bucharest Ilfov (BI)	1	6
South Muntenia (SM)	6	1
South West Oltenia (SW)	5	5
West (W)	6	4
South East (SE)	9	2
North West (NW)	10	2
Center (C)	9	2
North East (NE)	6	4

Source: developed by authors.

## References

[1] Eurostat, Data Portal. European Commission, Luxembourg. https://ec.europa.eu/eurostat/data/database.

[2] The World Bank, DataBank Health Nutrition and Population Statistics: Population Estimates and Projections. https://databank.worldbank.org/source/population-estimates-and-projections.

[3] World Health Organization (2020). Romania: Country Case Study on the Integrated Delivery of Long-Term Care.

[4] United Nations (2015). Transforming Our World: The 2030 Agenda for Sustainable Development.

[5] Institutul de Cercetare a Calității Vieții (2020). Calitatea Vieții Vârstnicilor. TENDINȚE și Riscuri în Contextul Pandemiei.

[6] Consiliul Național al Persoanelor Vârstnice (2019). Protectia Socială a Persoanelor Vârstnice si Drepturile Acestora.

[7] Cornea V. (2017). Institutional and Administrative Answers to the Phenomenon of Demographic Aging:(re) Configuration of the Social Services Infrastructure. Public Adm. Reg. Stud..

[8] Stănculescu S.M., Marin M. (2019). Dezvoltarea Serviciilor Sociale Adresate Persoanelor Vârstnice în România: Contribuţia Fondurilor Europene. Calit. Vieții.

[9] Ministerul Muncii si Solidaritatii Sociale HG Nr. 426 Din 27 Mai 2020 Privind Aprobarea Standardelor de Cost Pentru Serviciile Sociale. Monitorul Oficial Nr. 465 Din 2 Iunie 2020. https://mmuncii.ro/j33/images/Documente/Familie/2020/HG_426_2020_standarde_de_cost.pdf.

[10] Casa Nationala de Pensii Publice Statistici. https://www.cnpp.ro/en/indicatori-statistici-pilon-i.

[11] Ghenta M., Matei A., Mladen-Macovei L., Vasilescu M.D., Bobârnat E.-S. (2021). Sustainable Care and Factors Associated with Quality of Life among Older Beneficiaries of Social Services. Sustainability.

[12] Walker A. (2005). A European perspective on quality of life in old age. Eur. J. Ageing.

[13] Van Leeuwen K.M., van Loon M.S., Van Nes F.A., Bosmans J.E., de Vet H.C.W., Ket J.C.F., Widdershoven G.A.M., Ostelo R.W.J.G. (2019). What does quality of life mean to older adults? A thematic synthesis. PLoS ONE.

[14] Noll H.H. (2002). Towards a European system of social indicators: Theoretical framework and system architecture. Soc. Indic. Res..

[15] Bowling A., Brown J., Bowling A., Flynn T. (2004). A taxonomy and overview of quality of life. Models of Quality of Life: A Taxonomy and Systematic Review of the Literature.

[16] Tesch-Römer C., von Kondratowitz H.J., Motel-Klingebiel A., Daatland S.O., Herlofson K. (2001). Quality of life in the context of intergenerational solidarity. Ageing, Intergenerational Relations, Care Systems and Quality of Life.

[17] Gabriel Z., Bowling A., Walker A., Hagan Hennessy C. (2004). Quality of life in old age from the perspectives of older people. Growing Older: Quality of Life in Old Age.

[18] Gabriel Z., Bowling A. (2004). Quality of life from the perspectives of older people. Ageing Soc..

[19] Nazroo J., Bajekal M., Blane D., Grewal I., Walker A., Hagan Hennessy C. (2004). Ethnic inequalities. Growing Older: Quality of Life in Old Age.

[20] Fernandez-Ballesteros R. (1998). Quality of life: The differential conditions. Psychol. Spain.

[21] Tester S., Hubbard G., Downs M., MacDonald C., Murphy J., Walker A., Hagan Hennessy C. (2004). Frailty and institutional life. Growing Older: Quality of Life in Old Age.

[22] Lee D.T., Yu D.S., Kwong A.N. (2009). Quality of life of older people in residential care home: A literature review. J. Nurs. Healthc. Chronic Illn..

[23] Duncan-Myers A.M., Huebner R.A. (2000). Relationship between choice and quality of life among residents in long-term-care facilities. Am. J. Occup. Ther..

[24] Tu Y.C., Wang R.H., Yeh S.H. (2006). Relationship between perceived empowerment care and quality of life among elderly residents within nursing homes in Taiwan: A questionnaire survey. Int. J. Nurs. Stud..

[25] Tseng S.Z., Wang R.H. (2001). Quality of life and related factors among elderly nursing home residents in southern Taiwan. Public Health Nurs..

[26] Bowling A. (2010). Do older and younger people differ in their reported wellbeing? A national survey of adults in Britain. Fam. Pract..

[27] Rafnsson S.B., Shankar A., Steptoe A. (2015). Longitudinal influences of social network characteristics on subjective well-being of older adults: Findings from the ELSA study. J. Aging Health.

[28] Siette J., Jorgensen M.L., Georgiou A., Dodds L., McClean T., Westbrook J.I. (2021). Quality of life measurement in community-based aged care: Understanding variation between clients and between care service providers. BMC Geriatr..

[29] Hill R. Linking quality of home and community-based care and quality of life in frail older adults. Proceedings of the Paper Presented at the 54th Annual Scientific Meeting of the Gerontological Society of America.

[30] Lamb G.S., Noelker L.S., Harel Z. (2001). Assessing quality across the care continuum. Linking Quality of Long-Term Care and Quality of Life.

[31] Kane R.A. (2001). Long-term care and a good quality of life: Bringing them closer together. Gerontologist.

[32] Kelley-Gillespie N. (2009). An Integrated Conceptual Model of Quality of Life for Older Adults Based on a Synthesis of the Literature. Appl. Res. Qual. Life.

[33] Noelker L.S., Harel Z., Noelker L.S., Harel Z. (2001). Humanizing long-term care: Forging a link between quality of care and quality of life. Linking Quality of Long-Term Care and Quality of Life.

[34] Al Ghassani A., Rababa M. (2021). Factors Associated with Home Care Outcomes among Community-Dwelling Older Adult Patients with Dementia. Dement. Geriatr. Cogn. Disord. Extra.

[35] Kiik S.M., Nuwa M.S. (2020). Quality of life of the elderly: A comparison between community-dwelling elderly and in social welfare institutions. Medisains.

[36] Telenius E.W., Engedal K., Bergland A. (2013). Physical performance and quality of life of nursing-home residents with mild and moderate dementia. Int. J. Environ. Res. Public Health.

[37] Nikmat A.W., Hawthorne G., Al-Mashoor S.H. (2015). The comparison of quality of life among people with mild dementia in nursing home and home care-a preliminary report. Dementia.

[38] Wysocki A., Butler M., Kane R.L., Kane R.A., Shippee T., Sainfort F. (2012). Long-Term Care for Older Adults: A Review of Home and Community-Based Services Versus Institutional Care. Comp. Eff. Rev..

[39] De Medeiros M.M.D., Carletti T.M., Magno M.B., Maia L.C., Cavalcanti Y.W., Rodrigues-Garcia R.C.M. (2020). Does the institutionalization influence elderly’s quality of life? A systematic review and meta-analysis. BMC Geriatr..

[40] Simeão S.F.A.P., Martins G.A.L., Gatti M.A.N., Conti M.H.S., Vitta A., Marta S.N. (2018). Comparative study of quality of life of elderly nursing home residents and those attneding a day Center. Cienc. Saude Coletiva.

[41] Bryceson D., Vuorela U., Bryceson D., Vuorela U. (2002). Transnational Families in the Twenty First Century. The Transnational Family: New European Frontiers and Global Networks.

[42] Zontini E. (2004). Immigrant women in Barcelona: Coping with the consequences of transnational lives. J. Ethn. Migr. Stud..

[43] Czapka E.A., Sagbakken M. (2020). Challenges related to providing care for parents with dementia across borders: A qualitative study on transnational carers in Oslo. J. Aging Stud..

[44] Krzyżowski Ł. (2015). Social remittances and modifications of Polish intergenerational care cultures. Polish migrants in Austria and Iceland and their elderly parents. Studia Socjol..

[45] Saraceno C. (2016). Varieties of familialism: Comparing for Southern European and East Asian welfare regimes. J. Eur. Soc. Policy.

[46] Wilding R., Baldassar L. (2009). Transnational family-work balance. J. Fam. Stud..

[47] Sun K.C. (2012). Fashioning the reciprocal norms of elder care: A case of immigrants in the United States and their parents. Taiwan J. Fam. Issues.

[48] Hyde M., Wiggins R.D., Higgs P., Blane D. (2003). A measure of quality of life in early old age: The theory, development and properties of a needs satisfaction model (CASP-19). Aging Ment. Health.

[49] Montuclard L., Garrouste-Orgeas M., Timsit J.F., Misset B., De Jonghe B., Carlet J. (2000). Outcome, functional autonomy, and quality of life of elderly patients with a long-term intensive care unit stay. Crit. Care Med..

[50] Dahlberg L., McKee K.J. (2014). Correlates of social and emotional loneliness in older people: Evidence from an English community study. Aging Ment. Health.

[51] Schenk L., Meyer R., Behr A., Kuhlmey A., Holzhausen M. (2013). Quality of life in nursing homes: Results of a qualitative resident survey. Qual. Life Res..

[52] Barbour R.S., Kitzinger J. (1999). Developing Focus Group Research: Politics, Theory and Practice.

[53] Tonon G., Tonon G. (2015). Relevance of the Use of Qualitative Methods in the Study of Quality of Life. Qualitative Studies in Quality of Life.

[54] Rodriguez de la Vega L., Tonon G. (2015). The Role of Context and Culture in Quality of Life Studies. Qualitative Studies in Quality of Life.

[55] Malterud K., Siersma V.D., Guassora A.D. (2016). Sample Size in Qualitative Interview Studies: Guided by Information Power. Qual. Health Res..

[56] Precupetu I., Aartsen M., Vasile M. (2019). Social Exclusion and Mental Wellbeing in Older Romanians. Soc. Incl..

[57] Ritchie J., Lewis J., Elam G. (2003). Designing and Selecting Samples. Qualitative Research Practice.

[58] Braun V., Clarke V. (2006). Using thematic analysis in psychology. Qual. Res. Psychol..

[59] Bernard H., Wutich A., Ryan G. (2017). Analyzing Qualitative Data: Systematic Approaches.

[60] Murphy K., Cooney A., Casey D. (2014). Improving the quality of life for older people in long-term care settings. J. Comp. Eff. Res..

[61] Carr A.J., Gibson B., Robinson P.G. (2001). Is quality of life determined by expectations or experience?. BMJ.

[62] Holzhausen M., Kuhlmey A., Martus P. (2010). Individualized measurement of quality of life in older adults: Development and pilot testing of a new tool. Eur. J. Ageing.

[63] Soósová M.S. (2016). Determinants of quality of life in the elderly. Cent. Eur. J. Nurs. Midw..

[64] Dixe M.d.A.C.R., da Conceição Teixeira L.F., Areosa T.J.T.C.C., Frontini R.C., Peralta T.J.A., Querido A.I.F. (2019). Needs and skills of informal caregivers to care for a dependent person: A cross-sectional study. BMC Geriatr..

[65] Lightfoot E., Moone R.P. (2020). Caregiving in Times of Uncertainty: Helping Adult Children of Aging Parents Find Support during the COVID-19 Outbreak. J. Gerontol. Soc. Work.

[66] Phillips D., Paul G., Fahy M., Dowling-Hetherington L., Kroll T., Moloney B., Duffy C., Fealy G., Lafferty A. (2020). The invisibleworkforce during the COVID-19 pandemic: Family carers at the frontline. HRB Open Res..

[67] Nikmat A.W., Al-Mashoor S.H., Hashim N.A. (2015). Quality of life in people with cognitive impairment: Nursing homes versus home care. Int. Psychogeriatr..

[68] Harrad R., Sulla F. (2018). Factors associated with and impact of burnout in nursing and residential home care workers for the elderly. Acta Biomed..

[69] McHugh M.D., Kutney-Lee A., Cimiotti J.P., Sloane D.M., Aiken L.H. (2011). Nurses’ widespread job dissatisfaction, burnout, and frustration with health benefits signal problems for patient care. Health Aff..

[70] Trukeschitz B., Hajji A., Kieninger J., Malley J., Linnosmaa I., Forder J. (2021). Investigating factors influencing quality-of-life effects of home care services in Austria, England and Finland: A comparative analysis. J. Eur. Soc. Policy.

[71] Lao S., Low L., Wong K. (2019). Older residents’ perceptions of family involvement in residential care. Int. J. Qual. Stud. Health Well-Being.

[72] Bosch-Farré C., Malagón-Aguilera M.C., Ballester-Ferrando D., Bertran-Noguer C., Bonmatí-Tomàs A., Gelabert-Vilella S., Juvinyà-Canal D. (2020). Healthy Ageing in Place: Enablers and Barriers from the Perspective of the Elderly. A Qualitative Study. Int. J. Environ. Res. Public Health.

